# The beneficial effect of testing: an event-related potential study

**DOI:** 10.3389/fnbeh.2015.00248

**Published:** 2015-09-17

**Authors:** Cheng-Hua Bai, Emma K. Bridger, Hubert D. Zimmer, Axel Mecklinger

**Affiliations:** ^1^Experimental Neuropsychology Unit, Department of Psychology, Saarland UniversitySaarbrücken, Germany; ^2^Division of Psychology, Birmingham City UniversityBirmingham, UK; ^3^Brain and Cognition Unit, Department of Psychology, Saarland UniversitySaarbrücken, Germany

**Keywords:** episodic memory, testing effect, ERP, reinstatement, memory retrieval

## Abstract

The enhanced memory performance for items that are tested as compared to being restudied (the testing effect) is a frequently reported memory phenomenon. According to the episodic context account of the testing effect, this beneficial effect of testing is related to a process which reinstates the previously learnt episodic information. Few studies have explored the neural correlates of this effect at the time point when testing takes place, however. In this study, we utilized the ERP correlates of successful memory encoding to address this issue, hypothesizing that if the benefit of testing is due to retrieval-related processes at test then subsequent memory effects (SMEs) should resemble the ERP correlates of retrieval-based processing in their temporal and spatial characteristics. Participants were asked to learn Swahili-German word pairs before items were presented in either a *testing* or a *restudy* condition. Memory performance was assessed immediately and 1-day later with a cued recall task. Successfully recalling items at test increased the likelihood that items were remembered over time compared to items which were only restudied. An ERP subsequent memory contrast (later remembered vs. later forgotten tested items), which reflects the engagement of processes that ensure items are recallable the next day were topographically comparable with the ERP correlate of immediate recollection (immediately remembered vs. immediately forgotten tested items). This result shows that the processes which allow items to be more memorable over time share qualitatively similar neural correlates with the processes that relate to successful retrieval at test. This finding supports the notion that testing is more beneficial than restudying on memory performance over time because of its engagement of retrieval processes, such as the re-encoding of actively retrieved memory representations.

## Introduction

The testing effect refers to those findings which indicate that testing a studied list leads to better memory performance in a final test than restudying the list. In other words, testing is not only an assessment of memory, but also a way to change memory and consequently researchers have suggested that testing should be used as an efficient learning strategy (for review, see Roediger and Karpicke, 2006; Karpicke et al., 2014). The majority of experiments on this topic are behavioral studies, whilst the neuronal underpinning of the testing benefit has been addressed only recently (Eriksson et al., 2011; van den Broek et al., 2013; Wing et al., 2013; Rosburg et al., 2015). In the current study, we explored learning-by-testing mechanisms by analyzing the electrophysiological correlates of successful encoding during testing using a subsequent memory paradigm.

The beneficial effect of testing on memory is thought to come about because of the consequences of repeatedly retrieving information (Roediger and Butler, 2011). Researchers often refer to this process as retrieval practice, which in turn is thought to be able to contribute to memory via several mechanisms. For example, more effort might be allocated to items during testing than during restudy, which leads to enhanced reprocessing of information (Pyc and Rawson, 2009). Additionally, according to the transfer appropriate processing account, similar processes initiated during testing as for those required in the final memory test make the material more accessible in the final test (McDaniel et al., 1989; McDaniel and Fisher, 1991). Furthermore, testing requires the retrieval of information from memory. Retrieval processes may cause semantically-related information to be generated in a way that elaborates the retrieval cue as well as strengthening the relation between cue and target (Carpenter and DeLosh, 2006; Carpenter, 2009, 2011). Encoded information consequently becomes easier to access during the final test (Bjork, 1975; McDaniel and Masson, 1985).

In a recent review, Karpicke et al. (2014) analyzed the conditions under which retrieval practice has shown a memory advantage compared to restudy. Several principles of retrieval-based learning were summarized in the episodic context account. The core assumption is that retrieval practice places participants into a retrieval mode in which they attempt to reconstruct the past and to reinstate the temporal context. This retrieval process causes the item to be updated within its context, such that it may become associated with multiple context cues after extended retrieval practice, making it more retrievable as a consequence. Karpicke et al. (2014) provide empirical evidence for this hypothesis by showing that source memory decisions during testing led to better performance in a final recall test than old-new recognition memory decisions, whereas both conditions were better than an elaboration (forming images or generating word associates) condition. According to most dual process models of recognition memory, two distinct retrieval processes serve recognition: an automatic familiarity process and a slower more effortful recollection process (Yonelinas et al., 2010). Critically, whereas both processes can support simple item recognition, only recollection can afford the reinstatement of an item in its context (e.g., Diana et al., 2007). If the effect of retrieval practice on later memory performance improvement is due to the reinstatement of the prior episodic context, neural correlates of recollection should be most pronounced at the time point when retrieval practice is actively engaged. Important to consider is that, although recollection may incidentally occur during restudy, it is likely to occur less often than during retrieval practice, because recollection is generally considered an effortful process (Jacoby, 1991, 1998) which requires the engagement of retrieval mode and is not explicitly demanded by the restudy task. Subsequently, expectations derived from the episodic context account would specify that recollection processes should be evident under retrieval practice conditions, yet negligible during restudy.

In line with the accounts reviewed above, functional neuroimaging studies have recently provided evidence for the notion that testing benefits memory performance by recruiting retrieval processes. These studies have all employed versions of the subsequent memory paradigm (Davachi et al., 2001; Paller and Wagner, 2002). After an initial study phase (Phase 1), items were either restudied or tested in Phase 2, and during this period brain activity was measured. A final test (Phase 3) followed after some duration to assess memory performance. To disclose processes during testing or restudy condition, and to relate it with successful memory performance in a later test, items presented in Phase 2 were sorted into those that were subsequently remembered or forgotten in Phase 3. Brain activity locked to remembered items is usually then contrasted with forgotten items in order to capture subsequent memory effects (SMEs) and determine whether they differ for the two conditions.

In one such study, Wing et al. (2013) asked participants to learn pairs of weakly associated nouns and then tested them on these a day later. In the testing condition, activity in the hippocampus, left middle temporal cortex and medial prefrontal cortex was larger to subsequently remembered than forgotten items. Additional connectivity analyses revealed increased coupling between the hippocampus and ventrolateral prefrontal cortex, medial pre-frontal cortex, and posterior cingulate cortex in the testing condition. The activation in the middle temporal lobe, especially the hippocampus is taken to reflect relational memory processes. Relational processes enable recollection by binding disparate information into coherent representations during retrieval. Hence, during retrieval practice these processes are taken to strengthen the previously learned word-word associations or generate new associations that provide additional retrieval cues and improve retention in the final memory test (Wing et al., 2013).

In another study, van den Broek et al. (2013) asked participants to learn the Dutch translations of Swahili words. Participants practiced all the items three times through three repeated restudy/testing blocks in Phase 2 and the final test followed after 1 week. Activity was greater in the inferior frontal gyrus, midbrain and ventral striatum in the testing compared to the restudy condition, which was taken as evidence for higher cognitive control and modulation of memory by striatal reward circuits during testing. Critically, activity in left inferior parietal and left middle temporal areas predicted recall in the final memory test in the testing but not the restudy condition. The activity in the left inferior parietal and left middle temporal areas were modulated by the amount of information retrieved with higher activity during testing of subsequently remembered than forgotten words. As both areas have been consistently found to be involved in successful memory retrieval (Diana et al., 2007; Vilberg and Rugg, 2008) or the allocation of attention to retrieved information (Cabeza et al., 2008; Hutchinson et al., 2014), this study provides additional support for the view that testing involves the reinstatement of a prior study context by enhancing recollective or relational processing.

These two fMRI studies thus provide general support for the retrieval account of the testing effect, in which testing should cause retrieval of prior encoded episodes and a reinstantiation of the item in its context. This update of the memory trace during testing may provide additional cues for the final memory test. Comparable electrophysiological evidence is scarce, however, and the current study was designed to address this gap in the literature. Electrophysiological data is likely to be useful for understanding the mechanisms underlying the testing effect not only because of its greater temporal resolution, but also because decades of work using the event-related potential (ERP) technique in recognition tests have revealed a family of old/new effects thought to map onto distinct retrieval processes (for reviews see Friedman and Johnson, 2000; Mecklinger, 2000; Rugg and Curran, 2007). One such effect is usually referred to as the left-parietal old/new effect. Behavioral conditions that modulate recollection also modulate the left-parietal old/new effect and this effect has been shown to correlate with recollection-based memory judgments in item and associative memory studies (Friedman and Johnson, 2000). The temporal resolution of the ERP technique allows us to capture a recollection process at the time when the items were tested and prior studies have shown that the left-parietal old/new effect, the ERP correlate of recollection is most pronounced between approximately 500 and 700 ms after stimulus onset (Friedman and Johnson, 2000; Rugg and Curran, 2007). This electrophysiological marker of recollection thus allows for the exploration of a core prediction derived from the episodic context account outlined above: that recollection processes occur disproportionately more in testing than restudy conditions and it is this process which is associated with superior downstream memory performance for retrieval practice. This would be demonstrated if the electrophysiological subsequent memory effect (SME) in the testing condition but not in the restudy condition, was found to resemble the left-parietal old/new index of recollection.

To date, few ERP recognition studies have explored the electrophysiological consequences of testing. Rosburg et al. (2015) examined ERP correlates of immediate one-time testing in a source memory task. After initial learning, half of the learned items were presented in a source memory test before all old items were presented within a final old/new test. This allowed items that were studied and tested to be contrasted with those which were only studied once. Testing led to better item and source memory as well as speeded reaction times. The left parietal old/new effect in the final test was enhanced for items that were tested immediately after first-time study. In earlier work, the left-parietal old/new effect has been shown to correlate with the amount of retrieved information (Vilberg et al., 2006). In line with this, Rosburg and colleagues' results were taken as a demonstration that the one-time testing allows more detailed information recollected from the prior study episode. Although the data reported by Rosburg and colleagues clearly show the downstream impact of testing on recognition processes in the form of a boosted left parietal old/new effect, the electrophysiological correlates of processes engaged at the time of testing in contrast to a restudy condition using a subsequent memory paradigm have not yet been reported.

Another ERP effect that is frequently reported in recognition memory studies is the late posterior negativity (LPN). The LPN is a late and posteriorly distributed ERP component that is observed mainly in source recognition studies (Johansson and Mecklinger, 2003). It onsets around the time recognition decisions are given and is thought to reflect the assessment and evaluation of information retrieved from memory in situations in which memory features cannot easily be recovered. The LPN is most pronounced when extended retrieval processing is required, for example in situations in which multi-featured memory traces have to be discriminated (Leynes and Kakadia, 2013) or when the to-be-discriminated memory traces are weak or overlapping (Rosburg et al., 2011).

In the current study, participants studied Swahili-German word pairs in Phase 1. During Phase 2, pairs were either tested or restudied whilst EEG was recorded. The final test (Phase 3) followed 1 day later to increase the likelihood of observing a substantial testing effect (Butler and Roediger, 2007; Karpicke and Roediger, 2008). The main prediction for the behavioral results was that pairs tested in Phase 2 should be better remembered in Phase 3 than pairs that were restudied in Phase 2. For the ERP results, we expected subsequent memory effects (SMEs) for both studied and tested pairs in Phase 2. However, it was predicted that the SME during testing should differ qualitatively from the SME during restudy if distinctive retrieval processes are engaged in the two conditions. If retrieval practice promotes learning by the recruitment of recollection-like processes we expected the SME for tested items to resemble the late parietal old/new effect, i.e., the putative ERP correlate of recollection. The ERP correlate of recollection was assessed by contrasting the ERP response to items correctly recalled in Phase 2 but not in Phase 3, with those elicited by items which were neither recalled in Phase 2 and Phase 3 (see Table 1). If the LPN is related to the assessment of retrieved information, it is hypothesized that the LPN will be observed in the testing condition in which extended retrieval processing is required but not in the restudy condition in which retrieval does not take place. At the same time, we also expected the LPN to covary with the testing conditions, with the largest LPN in those testing trials in which knowledge from a prior study episode is not readily recovered in either Phase 2 or in Phase 3 and thus for which it is assumed that the resulting evaluation demands are high.

**Table 1:** **Condition labels categorized by experimental conditions and Memory Condition at three time points**.

**Practice condition**	**Phase 2 practice**	**Day1 recall**	**Phase 3 Day2 recall**	**Condition label**
Restudy		R	R	SR
		R	F	SF
Testing	R	R	R	RR
	R	R	F	RF
	F	F	F	FF

*R, Remember; F, Forgotten; S, restudy*.

## Methods

### Participants

Twenty-six students enrolled at University of Saarland gave informed consent prior to participation. Participants were compensated with either course credit or cash (8€/h). An additional 10€were given to the top 25% performers based on their performance at final recall. All participants were right-handed (Oldfield, 1971), reported no history of neurological disorders and had normal or corrected vision. The study was approved by the Ethic Committee of the Social and Applied Human Sciences of Saarland University. Two participants did not participate in all sessions, three had very poor performance (less than 25% correct at Day 1 recall), one had already participated in a similar experiment, and five had to be excluded due to insufficient artifact-free trials for ERP analysis (<16 trials). Fifteen participants thus entered the final analysis (aged 20–28 years old, *M* = 22.87, *SD* = 2.00).

### Materials

Stimuli were 220 Swahili-German word-pairs for which the German words had a frequency of between 10 and 100 occurrences per million (Mannheim frequency per Million; Baayen et al., 1995). All words referred to touchable nouns. Swahili words were translations of the German target words. Where no equivalent translation was available from online dictionaries, a near-synonym or a related-norm was selected. Prenasalized consonants in Swahili (e.g., “mv”) which are difficult for German readers to pronounce were kept to a minimum. Word length was matched so that on average both Swahili and German words were 6-letters long. A complete list of stimuli is available in Supplementary Table 1.

### Design overview

**Figure 1:**
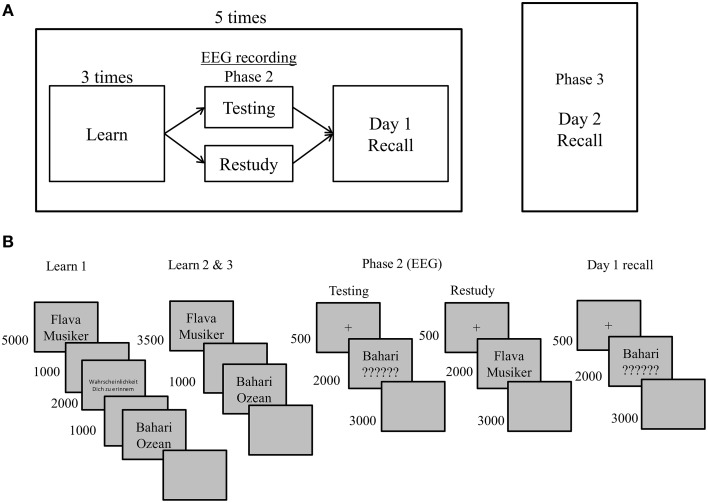
**(A)** Illustration of the procedure realized in each session. **(B)** Procedure for one cycle on Day 1. Five such cycles each consisting of 22 different items were run on Day 1. The procedure of the final cued recall test on Day 2 was identical to Day 1 recall except for a longer response deadline to 6000 ms and the testing of all 110 items, which is not illustrated in this figure. Note that in Phase 2 (the EEG session) participants did not respond before the offset of the restudy or testing cues which were presented for 2000 ms, respectively.

The experiment consisted of two sessions separated by 1 week. Each session comprised five cycles (each consisting of Phases 1 and 2) and a 2-day final recall (Phase 3). In each cycle participants studied 22 word-pairs. In the final test all 110 word-pairs studied on the previous day were tested (see Figure 1A). During the initial learning phase, word-pairs were presented three times in randomized order. Phase 2—during which EEG was recorded—followed initial learning. In Phase 2, 11 word-pairs were restudied again whereas the remaining 11 word-pairs of the study list were tested. Additionally, at the end of each cycle all 22 word-pairs were tested in a cued recall task (Day 1 recall). In this test, only Swahili words were presented as cues and participants had to retrieve the associated German words. Participants processed five of these study cycles on Day 1. Approximately 20~28 h later, participants returned for the final cued recall test (hereafter, Day 2 recall). To obtain sufficiently large trial numbers for the ERP analyses, the same procedure was repeated a week later with a different set of stimuli. In total, participants processed 110 items in the restudy and 110 items in the testing condition.

### Procedure

Each session began with the application of the electrode cap. All instructions were given both verbally and were shown on the computer screen at the beginning of the actual experiment. Participants began with a practice session comprising six word-pairs to familiarize them with the task procedure. As illustrated in Figure 1B, in each learning phase, word-pairs were presented in black against a gray background for 5000 ms on the display followed by a 1000 ms blank screen. Participants were encouraged to memorize word-pairs during this time. Participants were asked to judge how likely it was that they would remember the word-pair after the first presentation of each word-pair. They were instructed to use the right index finger to make a judgment of learning (JOL) on a 5-step scale where 1 means “definitely forget,” 2 “probably forget,” 3 “unsure,” 4 “probably remember,” and 5 “definitely remember” (Skavhaug et al., 2010). This judgment was given when “Wahrscheinlichkeit Dich zu erinnern” (“likelihood that you will remember”) was displayed. The JOL trial terminated when an answer was given or after 2000 ms, and was followed by a 1000 ms blank screen. The JOL data will not be reported here. After initial learning trials were completed for all 22 word-pairs within a cycle, participants studied the same list of word-pairs two more times in randomized order, but no JOL was required for second and third learning presentations. The presentation time of the word-pairs was 3500 ms followed by a 1000 ms blank screen.

In Phase 2, 50% of the word-pairs were presented in the testing condition whilst the remainder of the pairs was restudied. The assignment of items to testing/restudy condition was counterbalanced across participants. In the testing condition, participants saw Swahili words above six question marks for 2000 ms and were required to recall the German words. At the offset of the stimuli, they were required to say the German translation for the Swahili word aloud within the 3000 ms deadline. In the restudy condition, participants saw the Swahili-German pairs for a fourth time for 2000 ms. Participants were required to say the German words aloud once the stimuli were removed from the screen within the 3000 ms deadline. Testing and restudy trials were blocked to minimize task-switching demands and the order of testing vs. restudy trials was counterbalanced across participants. At the end of each cycle, a Day 1 cued recall task was completed for the 22 word-pairs. Times of presentation and response requirements were identical to testing condition trials. Participants took a self-paced break and proceeded to the next cycle. Each session took approximately 1 h.

Approximately 20~28 h later, participants returned to complete Day 2 cued recall test where all the 110 word pairs from the preceding day were tested. Each trial began with a 500 ms fixation cross and a 2000 ms presentation time with Swahili cue word and six question marks. Afterwards, participants had 6000 ms to provide a response for each Swahili word cue. The task lasted approximately 20 min. All responses were recorded via a microphone throughout. Correct and incorrect responses were coded online by an experimenter. No EEG was recorded during the final test.

### EEG acquisition and analysis

Fifty-eight Ag/AgCI electrodes were embedded in an elastic cap (Easycap, Herrsching, Germany) based on the extended international 10–20 system. The electroencephalogram (EEG) was recorded continuously with a sampling rate of 500 Hz. Two additional pairs of electrodes were used: one pair was placed on the outer canthi for horizontal EOG. Another two electrodes were placed above and below the right eye for vertical EOG, respectively. An electrode placed anterior to Fz served as the ground. EEG was referenced online to the left mastoid. The impedances of the recording electrodes were kept below 5 kΩ. Data was recorded online and processed offline by commercial software Brain Vision Recorder and Analyzer (Brain Products). EEG signals were recorded with a digital bandpass filter (DC-70 Hz) at a rate of 500 Hz with an extra filter applied offline (0.03–30 Hz, 12 dB/oct). Final epochs extended from 100 ms prestimulus until 1000 ms after stimulus presentation during Phase 2. Data were downsampled to 250 Hz and offline re-referenced to the average of the mastoid signals. Baseline correction started from 100 ms before stimulus onset to stimulus onset. A correction algorithm based on independent component analysis (ICA) was employed for EOG artifact rejection (Makeig et al., 2004).

ERP waveforms were created for five conditions (see Table 1). ERPs to restudied items are labeled as “studied later-remembered (SR)” or “studied later-forgotten (SF)” pairs, depending upon whether they were recalled correctly on Day 2. Tested items were separated into three categories. Tested items recalled correctly at Phase 2 and on Day 2 were labeled as “remember-remember (RR)”; tested items recalled correctly at Phase 2, but forgotten on Day 2 are labeled as “remember-forgotten (RF)”; and tested items which were not correctly retrieved at either Phase 2 or on Day 2 are labeled as “forgotten-forgotten (FF).” The mean number and range (in parenthesis) of trials entering into each individual's average were as follows: 35 (16–53) SR; 39 (26–51) SF; 37 (21–57) RR; 30 (16–43) RF; 34 (16–54) FF.

ERP analyses are based on the following contrasts: (i) the SME for restudied items was revealed by contrasting SR and SF; (ii) the SME for tested items was revealed by contrasting RR and RF; (iii) the ERP correlate of immediate-retrieval was assessed by contrasting RF and FF which should isolate immediate retrieval success during Phase 2 for tested items. The comparison between this contrast and the SME for tested items [contrast (ii)] in the 500–700 ms time interval in which the ERP correlate of recollection can reliably be recorded was used to test whether correct recall on Day 2 is associated with the ERP correlate of recollection. The fourth contrast (iv) was between ERPs to studied items (collapsed across SR and SF) and ERPs to RR, RF, and FF pairs, to test whether the LPN is elicited solely by tested items and if so, whether it is modulated by the ease with which memory representations are retrievable. This fourth contrast was specifically tested in the last time window 700–1000 ms due to the fact that the onset of LPN found in previous studies is usually later than the time window of recollection.

ANOVAs were used to test mean amplitude differences for each condition (i.e., SR, SF; RR, RF, FF) from three selected time windows: 300–500, 500–700, and 700–1000 ms. These time windows were chosen because the effects of interest were present in the time intervals and because they correspond with those time intervals used for the conventional analysis of ERP memory encoding and retrieval studies. The 300–500 ms window covers that in which an early mid-frontal old/new effect often associated with familiarity is usually reported. The 500–700 ms time window was selected to capture the left-parietal old/new effect. Additionally, the LPN is usually not observed before 700 ms after onset of a retrieval cue and extends for several 100 ms (Johansson and Mecklinger, 2003) and thus the 700–1000 ms time window was used to capture this effect. Consistent with other ERP studies exploring encoding and retrieval processes (Bader et al., 2010; Halsband et al., 2012), mean amplitudes were taken from three frontal (F3, Fz, F4), three central (C3, Cz, C4), and three parietal (P3, Pz, P4) electrodes, chosen because they best represent the scalp and should allow detection of frontally-extended SMEs as well as the left-parietal effect and the LPN. Repeated measures ANOVAs included the factors Memory Condition (see Result Section for the factors levels used in the four contrasts) and 3 Anterior-Posterior (frontal, central, parietal) and 3 Laterality (left [3], middle [z], right [4]). Degrees of freedom were adjusted for the ANOVAs by incorporating the Greenhouse-Geisser correction for violations of sphericity when appropriate. The adapted degrees of freedom are reported for both behavioral and ERP data.

## Results

### Behavioral data

**Figure 2:**
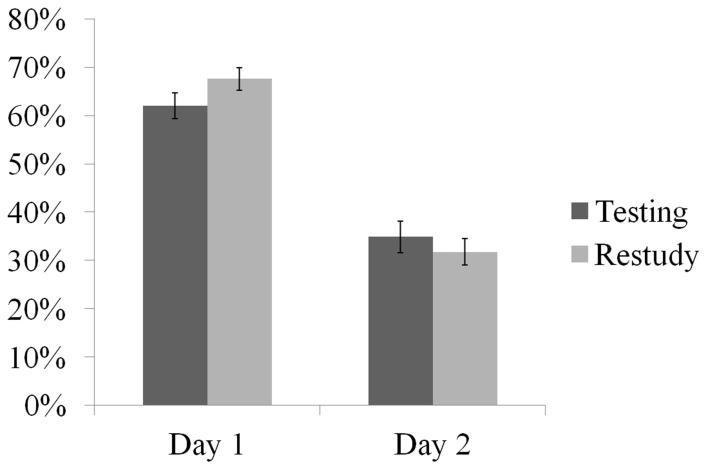
**Percent correct for tested and restudied items at cued recall on Day 1 and at final recall on Day 2**. Error bars show 1 ± standard error mean.

Figure 2 shows mean proportions of correct recall for the testing/restudy conditions on Day 1 and Day 2. As revealed by a Shapiro-Wilk test for normality (Shapiro and Wilk, 1965) all four mean proportion scores were normally distributed (*p*>0.10). An ANOVA with factors testing/restudy condition and time (Day 1, 2 recall) revealed a main effect of time, *F*_(1, 14)_ = 298.90, *p* < 0.01 and an interaction between testing/restudy conditions and time, *F*_(1, 14)_ = 33.39, *p* < 0.01. To follow up the interaction effect, we compared the amount of recalled items between testing and restudy conditions on Day 1 and Day 2, respectively. The result showed that on Day 1 more restudied items (*M* = 0.68, *SD* = 0.13) were recalled than tested items (*M* = 0.62, *SD* = 0.10), *t*_(14)_ = −2.31, *p* < 0.05, while on Day 2 this difference was reversed. A marginally significant testing effect was found on Day 2 where participants were able to recall more tested items (*M* = 0.35, *SD* = 0.09) than restudied items (*M* = 0.32, *SD* = 0.11), *t*_(14)_ = 1.79, *p* = 0.10. In addition, the difference in the amount of correctly recalled items from Day 1 to Day 2 is significantly smaller in testing (Mean difference from Day 1 to Day 2 = 0.27, *SD* = 0.06) than in the restudy condition (Mean difference from Day 1 to Day 2 = 0.36, *SD* = 0.09), *t*_(14)_ = −5.78, *p* < 0.01. This suggests that, once successfully recalled in Phase 2, tested items were less likely to be forgotten on Day 2 in comparison to merely restudied items. This benefit of testing from Day 1 to Day 2 recall allowed us to proceed with the ERP analysis to explore the neural underpinnings of this behavioral testing effect and its relevance on later memory performance as presented in the following.

### ERP data

#### Restudy condition

**Figure 3:**
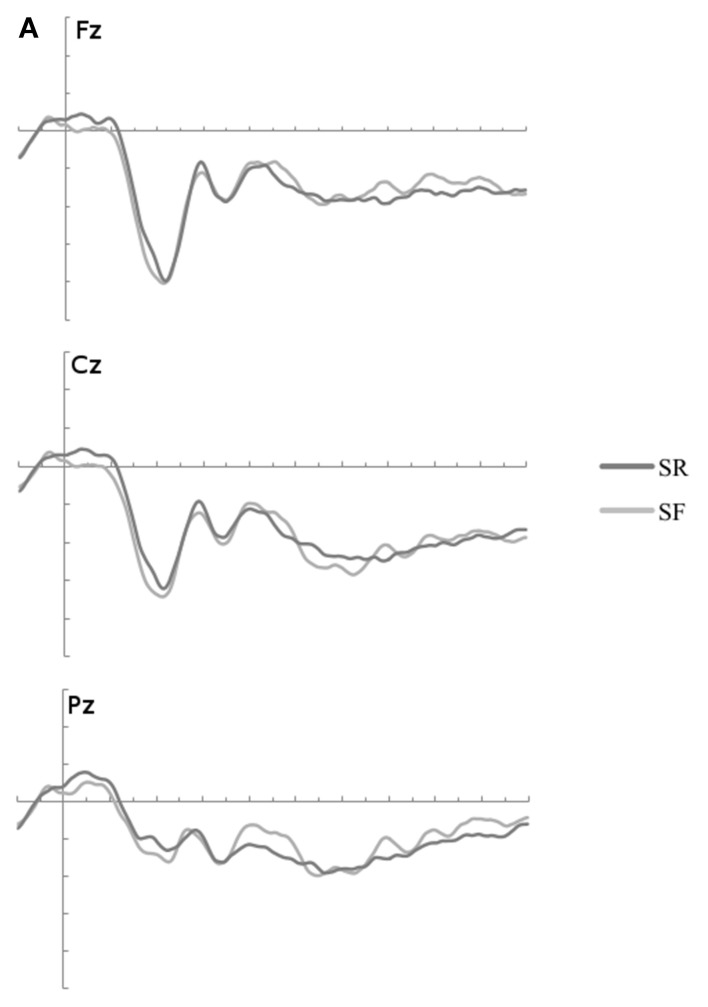

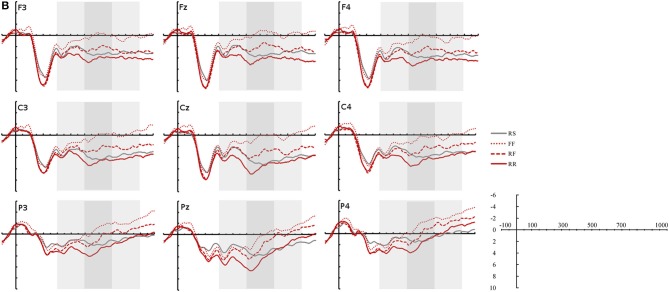
**(A)** The ERP waveforms to restudied items which were later remember and forgotten (SR/SF) were not significantly different at any time windows of interest. **(B)** ERP waveforms to all restudied items (RS) and tested items categories by Memory Condition (RR, RF, FF). ERPs are plotted from 100 ms before stimulus onset to 1000 ms thereafter at frontal, central and posterior midline sites: Fz, Cz, and Pz. Three time windows of interest are marked in gray. The waveforms were low-passed filtered at 12 Hz for illustration. RR, remembered; RF, later forgotten; FF, immediately forgotten.

This analysis compared ERPs elicited by restudied items that were either remembered or forgotten on Day 2 recall [contrast (i): Restudy SME]. As shown in Figure 3A, small differences from 300 to 500 ms at posterior sites were observed; however, a global ANOVA with the factors Memory Condition (SR/SF: later remembered/later forgotten) × 3 AP × 3 Laterality in the three selected time windows did not reveal any main effect of Memory Condition nor any interaction effect including this factor (See Table 2A). There were thus no significant ERP differences in the restudy condition of Phase 2 between items that were remembered or forgotten on the Day 2 recall test. Given this null effect and to make the remainder of the analyses more accessible, the two restudy conditions (SR/SF) were collapsed into one RS condition for the remainder of the relevant analyses.

**Table 2:** **ANOVA table for (A) Restudy SME, Test SME, and Immediate retrieval effect (B) LPN analyses**.

**(A)**					**(B)**		
**Contrast**	**Effect**	**300–500**	**500–700**	**700–1000**	**Contrast**	**Effect**	**700–1000**
(i)					(iv)		
Restudy subsequent memory effect					LPN		
SR/SF	Condition	n.s.	n.s.	n.s.	RS/RR	Condition	n.s.
	… × AP	n.s.	n.s.	n.s.		… × AP	*F*_(1.26, 17.61)_ = 4.33, *p* < 0.05
	… × LAT	n.s.	n.s.	n.s.		… × LAT	n.s.
	… × AP × LAT	n.s.	n.s.	n.s.		… × AP × LAT	n.s.
(ii)					RS/RF	Condition	n.s.
Test subsequent memory effect						… × AP	n.s.
RR/RF	Condition	*F*_(1, 14)_ = 5.35, *p* < 0.05	*F*_(1, 14)_ = 14.26, *p* < 0.01	*F*_(1, 14)_ = 6.21, *p* < 0.05		… × LAT	n.s.
	… × AP	n.s.	n.s.	n.s.		… × AP × LAT	n.s.
	… × LAT	n.s.	n.s.	n.s.	RS/FF	Condition	*F*_(1, 14)_ = 6.67, *p* < 0.05
	… × AP × LAT	n.s.	n.s.	n.s.		… × AP	n.s.
(iii)						… × LAT	n.s.
Immediate retrieval effect						… × AP × LAT	n.s.
RF/FF	Condition	n.s.	*F*_(1, 14)_ = 6.74, *p* < 0.05	n.s.	RR/FF	Condition	*F*_(1, 14)_ = 9.11, *p* < 0.01
	… × AP	n.s.	n.s.	*F*_(1.31, 18.27)_ = 5.95, *p* < 0.05		… × AP	*F*_(1.36, 19.09)_ = 5.27, *p* < 0.05
	… × LAT	n.s.	n.s.	n.s.		… × LAT	*F*_(1.31, 18.38)_ = 4.19, *p* < 0.05
	… × AP × LAT	n.s.	n.s.	n.s.		… × AP × LAT	*F*_(4, 56)_ = 3.69, *p* < 0.01

*Degrees of freedom, F- and p- values are listed only for significant results (p < 0.05). Anterior-posterior (AP), laterality (LAT). SR, studied remembered; SF, studied forgotten; RR, remembered; RF, later forgotten; FF, immediately forgotten. Non-significant is abbreviated as n.s. Shading indicates significant outcomes*.

#### Testing condition

Corresponding degrees of freedom, *F*- and *p*-values for contrasts related to the items in the testing condition are reported in Table 2.

##### SME for tested items

**Figure 4:**
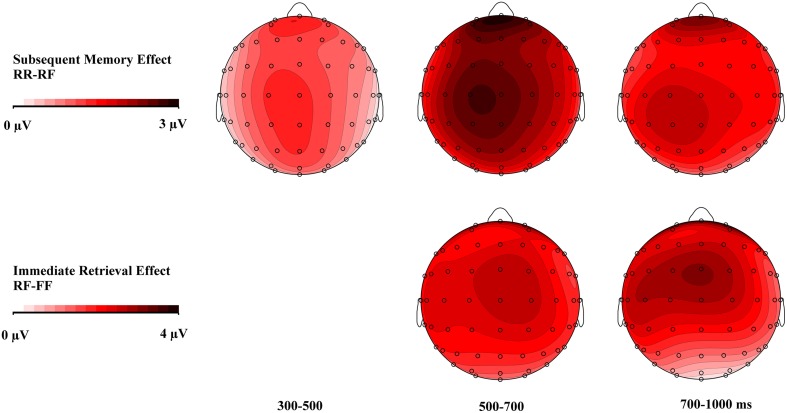
**Topographical maps showing the scalp distributions of the subsequent memory effect (RR-RF) and the immediate retrieval effect (RF-FF)**. The subsequent memory effect started at an earlier time window (300–500 ms). Both effects show similar scalp topography in the 500–700 ms time window. The voltage scale is from 0 to 3 μV for the subsequent memory condition and from 0 to 4 μV for the immediate retrieval condition.

As shown in Figure 3B, the ERPs to RR items start to diverge from ERPs to RF items around 300 ms post-stimulus, with a greater relative positivity for RR items. This difference is widely distributed across the scalp (see Figure 4 upper panel). A global ANOVA with factors of Memory Condition (RR/RF) × 3 AP × 3 Laterality revealed a main effect of subsequent memory in all three time windows of interest from 300–500, 500–700 to 700–1000 ms. No interaction between the Memory Condition factor and other factors was found in either time interval.

##### ERP correlates of immediate-retrieval

The waveforms to RF and FF in Figure 3B show that the ERPs to immediately-remembered items were more positive-going than to forgotten items. Even though visual inspection of the waveforms suggest that the waveforms for RF and FF begin to diverge at round 300 ms, no reliable effect involving the Memory Condition factor were found in the early (300–500 ms) time interval (see Table 2). Reliable differences between the RF and FF condition, however, start at 500 ms and on the basis of visual inspection (Figure 4 lower panel), this effect appears to be more frontal-central than posterior, particularly in the late time window (700–1000 ms). ANOVAs with factors of Memory Condition (RF/FF) × 3 AP × 3 Laterality revealed a main effect of Memory Condition in the 500–700 ms time window. In the 700–1000 ms time window, there was an interaction between Memory Condition and AP. Bonferroni adjusted follow-up tests (with the critical α-level set to *p* = 0.02) with factors of Memory Condition (RF/FF) for each of the 3 levels of AP (frontal, central or posterior) revealed that the main effect of Memory Condition was not significant neither at frontal (*p*>0.04) nor at central (*p* < 0.05) or posterior sites (*p* < 0.21).

##### Comparing the SME and immediate-retrieval effect

Our main prediction was that if retrieval practice promotes learning by the recruitment of recollection-like processes, the SME for tested items should resemble the ERP correlate of immediate retrieval (as reflected in the RF/FF contrast). To directly test this, we examined whether the immediate-retrieval effect and the SME in the 500–700 ms time window in which the ERP correlate of recollection can reliably be recorded differ in scalp topography, as would be expected if different neuronal circuitries have contributed to both effects. To improve the sensitivity of this contrast, all 58 recording sites were included in this analysis. An additional analysis was conducted on amplitude normalized mean values to ensure that any differences in scalp topography between the two conditions do not result from amplitude differences (McCarthy and Wood, 1985). The ANOVA with factors Memory condition (RR-RF; RF-FF) and recording site did not reveal a significant interaction, [non-scaled data: *F*_(57, 798)_ = 0.66, *p* = 0.98; scaled data: *F*_(57, 798)_ = 0.68, *p* = 0.97], suggesting that highly similar brain circuitries were active in the immediate-retrieval processes and the 500–700 ms proportion of the SME contrast.

#### Comparison of restudy and testing condition

##### All restudied items (RS) vs. one tested condition (RR or RF or FF)

As reported in Table 2B, in this set of contrasts we explored whether and how mnemonic processing in the testing condition is reflected in the LPN, a late onsetting ERP component elicited by retrieval cues when memory contents are searched and retrieved. We first contrasted the LPN in the study condition (RS; collapsed across later forgotten [SF] and later remembered [SR] trials) separately with the three testing conditions RR, RF, and FF using ANOVAs with factors testing/restudy condition × 3 AP × 3 Laterality in the 700–1000 ms time window. For the RS vs. RR contrast there was a testing/restudy × AP interaction. Follow-up ANOVAs were performed for each level of the AP factor. While no effects were obtained at the frontal and central recordings, for the parietal recordings there was a testing/restudy by LAT interaction [*F*_(2, 28)_ = 5.72, *p* < 0.01]. Although this interaction is difficult to interpret, it most likely reflects a tendency for more negative going waveforms in the RR condition at right parietal recordings. In the RS/RF contrast, there were no effects involving the testing/restudy condition. Rather, the RS/FF contrast revealed a main effect of testing/restudy condition which reflects the broadly distributed LPN in the FF as compared to the RS condition.

In a final contrast, we explored whether the LPN within the testing conditions is modulated by the ease with which information can be recovered by contrasting tested items which were not retrieved at practice or Day 2 (FF) with those that were retrieved at practice and at Day 2 (RR). This analysis revealed a main effect of testing/restudy condition, interactions between the condition factor and the two other factors, AP and LAT, as well as a three-way interaction. Tested separately for each of the Laterality by AP combinations, a larger LPN for the FF than the RR condition was obtained at all nine electrode sites. *Post-hoc* analyses estimating the effect size using Cohen's *d*-values revealed that the LPN is most pronounced at left middle-posterior C3 and P3 electrode sites (*d* > 0.9) and also middle-right central Cz and C4 (*d* > 0.8) electrodes.

Taken together the LPN analyses revealed a topographically widespread (RS vs. FF contrast) and a left to midline centro-parietally accentuated LPN (RR vs. FF contrast). The LPN was generally larger in the testing condition that in the restudy condition, in line with the assumption that retrieval processes and evaluation of retrieved information took place to a greater extent in the former condition. Notably, the largest LPN difference was obtained when the restudy condition was contrasted with the testing condition (FF) for which the presumed demands on evaluative processing were largest, because these items were not recallable on either Days 1 or 2. For this latter testing condition the LPN was also larger than in the testing condition, in which items were readily assessable at Day 1 and Day 2 (RR) presumably resulting in low evaluation demands.

## Discussion

Many studies have demonstrated that testing during learning enhances later memory performance. The episodic context account is one model of the underlying mechanisms thought to drive the testing effect (Lehman et al., 2014). The core concept of this account is that retrieval practice encourages a retrieval process which leads learners to recollect the episode during learning; consequently re-instantiating the episodic context, enhancing associative processing during testing and improving retention in the final recall test.

The current ERP study provides support for the retrieval account of the testing effect. On the behavioral level, the forgetting rate from Day 1 to Day 2 was lower for pairs that were tested on Day 1 than for restudied materials. We now turn to the analyses of the ERP data to explore the neural underpinnings of this effect.

### Restudy

Although SMEs were expected in both the restudy and testing condition, no such effect was observed in the restudy condition. We speculate that this is likely to be due to the inclusion of three learning blocks prior to the restudy condition in Phase 2 of the current design. The processes which predict later memory performance for the restudied items and which are typically seen in ERP SME contrasts (i.e., Paller and Wagner, 2002) could have occurred during any of the preceding learning blocks, rendering them unobservable during Phase 2. This jittering of the point at which encoding occurred is likely to have diluted the SME in the restudy condition. An alternative, yet related account for the absent SME effect arises from considering studies which demonstrated a reversed SME in ERPs to non-words as compared to the SME to real words (e.g., Otten et al., 2007) or from studies which did not obtain SME in conditions in which items were not semantically processed (Mecklinger and Müller, 1996). The potential implications of this therefore are that seeing a restudied word pair for the fourth time reduces the likelihood of engaging in semantic processing or explicit encoding processes that are usually observed for real words seen in standard SME paradigms. It is not possible to consider the likelihoods of specific mechanisms on the basis of these data alone. However, the absence of an SME in the restudy condition alongside the robust SME in the testing condition strongly supports the core assumption of the episodic context account that testing conditions uniquely elicit mnemonic processes, which confer benefits for later recall. In the following, it is argued that the current data strongly indicate that these processes include recollection.

### Testing

The first contrast between items in the testing condition revealed ERPs to later-remembered items (RR) that were more positive-going than ERPs to items that were later-forgotten (RF). This finding is in line with our prediction despite the fact that this effect was widely distributed across the scalp at all time windows from 300 up to 1000 ms after stimulus onset, which has an earlier onset than the predicted time window in which the neural correlations of recollection were often observed (Paller and Wagner, 2002).

The contrast between RF and FF was presumed to reflect immediate-retrieval processes. This contrast was significant in the time window from 500 to 700 ms in which the parietal old/new effect, the ERP correlate of recollection is usually found (Rugg and Curran, 2007). Although the ERP correlate of recollection has been reported to focus principally at parietal recording sites (Vilberg and Rugg, 2008), we find only a main effect of Memory condition in this specific time window. Previous studies have also found the parietal old/new effect to be larger and more widely distributed in free recall task than in recognition task (Paller et al., 1988). In addition, it is also conceivable that cues in a foreign language evoke processes additional to the recollection processes and this may have rendered the effect more widely distributed across the scalp.

Notably, when contrasting the SME and immediate retrieval effect between 500 and 700 ms, we find that the effects do not differ in scalp topography even when a highly sensitive measure for topographical differences including 58 scalp electrodes was used. Although null findings require particular caution in EEG, these data are in line with the engagement of qualitatively similar neural circuitry associated with recollective processing in the two cases. The current findings support the episodic context account, which assumes that the testing effect is likely to be driven by an engagement of recollection at testing. Recollection can bind multiple aspects to represent an episode and recollective processing during testing may have strengthened the previously formed word-word associations or may have produced novel associations that facilitated retrieval on Day 2. By this the current data provide electrophysiological support for the retrieval account of the testing effect and complement brain imaging studies exploring the testing effect using the SME paradigm (van den Broek et al., 2013; Wing et al., 2013). A second but not mutually exclusive possibility is that the amplitude of ERPs for tested items in the 500–700 ms time window was modulated by memory strength. Visual inspection of this effect reveals a gradient of increasing amplitude among the three testing conditions (RR > RF > FF) in this time window (Figure 3B). To explore this possibility, we conducted an additional *post-hoc* analysis of the amplitude differences across the three testing conditions in this time window. An ANOVA with three testing conditions and AP and Laterality as factors revealed a main effect of testing condition, *F*_(1.43, 19.99)_ = 13.18, *p* < 0.01. The ERPs become increasingly positive the better an item was remembered, with largest values for word pairs remembered on Day 2 (RR) intermediate values for pairs remembered on Day 1 only (RF) and smallest values for words which were already forgotten at practice (FF). This effect is consistent with the view that items with high memory strength are better recollected; meaning that more information is in an accessible state and more likely to be remembered at both days.

### LPN is associated with post-retrieval memory search

In the late (700–1000 ms) time interval, an LPN was obtained which was generally larger in the testing than in the restudy condition. Notably, these LPN differences between testing and restudy were only obtained when the restudy condition was contrasted with the testing condition (FF) that imposed the highest demands on post-retrieval evaluative processing. In this latter testing condition the LPN was also larger than in the testing condition in which items were readily assessable resulting in low evaluation demands (RR). This pattern of results is consistent with the view that the LPN is most pronounced in situations with a high need for continuous evaluation of memory bound information. Notably the current results are also consistent with a second account of the LPN, namely that it is modulated by the specificity with which memory is searched. In one illustrative source memory study (Leynes and Kakadia, 2013) participants were required to discriminate either between performed and watched actions or between performed and interrupted actions. There was a large LPN when performed and interrupted actions had to be discriminated, i.e., a condition with a high amount of overlapping features between both sources in which as a consequence only a few features were diagnostic for the source decision. The LPN in the latter study was much smaller when performed and watched actions had to be discriminated and the two sources could be discriminated on a simple 1st vs. 3rd person perspective. In this framework, it is conceivable that a cued recall test such as the one used in the current testing condition imposes high demands for a highly specific memory search, as it requires one to discriminate between phonologically and semantically overlapping words in order to identify the item originally paired with the cue word. The highly overlapping lexical features of the German-target words may have lowered memory strength and may have given rise to extended retrieval processing as reflected in the LPN, particularly in situations in which retrieval processing was unsuccessful.

### Caveats

Although a robust benefit of testing was demonstrated when the amounts of forgetting from Day 1 to Day 2 were compared between testing and restudy conditions, only a marginally significant difference between testing and restudy on Day 2 was observed. This could arise from a number of aspects of the current design. Unlike studies which controlled for learning success before retrieval practice took place (Karpicke and Roediger, 2008; van den Broek et al., 2013), in the current study, participants learned all word pairs three times before the critical manipulation was introduced. Given the large number of test items required to provide sufficient ERP trials, the relatively high task difficulty may have meant some items were not encoded sufficiently within the initial three learning blocks. For those items not learned during Phase 1, once they were assigned to the testing condition, there was a low-likelihood that the items would be recovered. In contrast, the unlearned items from Phase 1 would receive a fourth learning opportunity once they were assigned into the restudy condition (Bahrick and Hall, 1991; Jang et al., 2012). This restriction of the experimental design conveys a disadvantage for tested items over restudied items on Day 1 (Toppino and Cohen, 2009), because the latter are shown again during Phase 2.

Another aspect of the testing paradigm sounds a note of caution when interpreting the ERP testing effects as SME. We took the increasing positivity from RF to RR as a correlate of processes that support later retrieval and therefore as a SME. However, it is possible that any effect which influences ERPs during memory retrieval can be mistaken for encoding effects caused by testing itself. It is principally not possible to determine whether these ERP effects reflect more general differences in memory strength as assumed by the memory strength account or are consequences of different memory strengths caused by former encoding as assumed by the retrieval account. For example, there could have been general a priori differences in memory strength between RR and FF word pairs rendering the former easier to retrieve than the latter. If this was the case, the ERP effects would reflect an item selection bias. Due to the high number of word pairs that we used in the present study we do not believe that a selection bias was at work but this issue should be more directly addressed in further studies.

In summary, the current study provides a direct link between the neural correlates of the SME and of immediate retrieval at the time point when testing occurs in comparison to restudy condition. Our findings support the episodic context account that testing engages recollection and that enhanced recollective processes improve retention in a later cued recall task. A second possible explanation is that the higher memory strength is, the higher is recallability on a later recall task and this retrieval practice further enhances later memory on a delayed test. In addition, we also show that at a late stage of retrieval processing the LPN reflects a highly specific memory search presumably imposed by the overlapping features of the to-be-discriminated words during cued recall.

### Conflict of interest statement

The authors declare that the research was conducted in the absence of any commercial or financial relationships that could be construed as a potential conflict of interest.
