# Supplementary material for: The beneficial effect of testing: an event-related potential study
Source: Front Behav Neurosci. 2015 Sep 17;9:248. doi: 10.3389/fnbeh.2015.00248 (PMC4584999; doi:10.3389/fnbeh.2015.00248)
Supplement: Supplementary file 1 [file Table1.DOCX]

***Supplementary Material***

**The Beneficial Effect of Testing: An Event-Related Potential Study**

**Cheng-Hua Bai^1^*, Emma K. Bridger^1, 3^, Hubert Zimmer^2^ and Axel Mecklinger^1^**

^1^Experimental Neuropsychology Unit, Department of Psychology, Saarland University, Saarbrücken, Germany

^2^Brain and Cognition Unit, Department of Psychology, Saarland University, Saarbrücken, Germany

^3^ Division of Psychology, Birmingham City University, Birmingham, UK

***Correspondence:** Cheng-hua Bai, Department of Psychology, Saarland University, Uni Campus, 66123 Saarbrücken, Germany. c.bai@mx.uni-saarland.de

**Supplementary Table:**

List of experimental material (220 Swahili-German word pairs)

| ID | cue | target | ID | cue | target | ID | cue | target | ID | cue | target |
| --- | --- | --- | --- | --- | --- | --- | --- | --- | --- | --- | --- |
| 1 | Afisa | Offizier | 56 | Kamata | Klinke | 111 | Meli | Flotte | 166 | Sahani | Teller |
| 2 | Alama | Fahne | 57 | Kanisa | Kloster | 112 | Meneja | Leiter | 167 | Saidi | Hausmeister |
| 3 | Askari | Polizist | 58 | Karakana | Stahlwerk | 113 | Mfuko | Paket | 168 | Sakafu | Etage |
| 4 | Aya | Absatz | 59 | Karatasi | Papier | 114 | Mfumo | Wolle | 169 | Saruji | Beton |
| 5 | Bahari | Ozean | 60 | Katibu | Sekretär | 115 | Mfupa | Knochen | 170 | Sebuleni | Wohnzimmer |
| 6 | Bahasha | Umschlag | 61 | Keki | Waffel | 116 | Mgodi | Grube | 171 | Shaba | Kupfer |
| 7 | Baiskeli | Fahrrad | 62 | Kiatu | Sattel | 117 | Mhimili | Achse | 172 | Shajara | Tagebuch |
| 8 | Bango | Plakat | 63 | Kiazi | Kartoffel | 118 | Mhudumu | Kellner | 173 | Shayiri | Roggen |
| 9 | Bata | Ente | 64 | Kibanda | Hütte | 119 | Miako | Flamme | 174 | Shujaa | Sieger |
| 10 | Biri | Zigarre | 65 | Kidini | Nonne | 120 | Milima | Gebirge | 175 | Siagi | Butter |
| 11 | Bomba | Pfeife | 66 | Kidole | Finger | 121 | Mishumaa | Kerze | 176 | Sikio | Hörer |
| 12 | Breki | Bremse | 67 | Kifaa | Apparat | 122 | Mitaro | Graben | 177 | Simama | Tribüne |
| 13 | Bunduki | Pistole | 68 | Kifaru | Panzer | 123 | Miti | Linde | 178 | Simba | Löwe |
| 14 | Bustani | Garten | 69 | Kijitabu | Broschüre | 124 | Mizigo | Gepäck | 179 | Simu | Telegramm |
| 15 | Chajio | Restaurant | 70 | Kikombe | Pokal | 125 | Mkazi | Einwohner | 180 | Sinema | Kino |
| 16 | Chama | Verband | 71 | Kilima | Hügel | 126 | Mkulima | Bauer | 181 | Skeli | Waage |
| 17 | Cheti | Urkunde | 72 | Kimbilio | Bunker | 127 | Mkurugenzi | Direktor | 182 | Sungura | Hase |
| 18 | Choma | Feuer | 73 | Kipando | Traktor | 128 | Moshi | Sauna | 183 | Sura | Fassade |
| 19 | Chuja | Stiefel | 74 | Kisiwa | Insel | 129 | Mtaalam | Experte | 184 | Suruali | Hose |
| 20 | Chuma | Eisen | 75 | Kitabu | Bibel | 130 | Mtoto | Baby | 185 | Suti | Anzug |
| 21 | Chumba | Schlafzimmer | 76 | Kiti | Sessel | 131 | Mtumishi | Diener | 186 | Taji | Krone |
| 22 | Chuo | Hochschule | 77 | Kiwanda | Fabrik | 132 | Mvua | Regen | 187 | Tariki | Fahrbahn |
| 23 | Chupa | Flasche | 78 | Kocha | Trainer | 133 | Mvulana | Junge | 188 | Tembe | Pille |
| 24 | Degaga | Brille | 79 | Kofia | Mütze | 134 | Mvuvi | Fischer | 189 | Teski | Taxi |
| 25 | Dereva | Reiter | 80 | Kombora | Bombe | 135 | Mwamba | Gestein | 190 | Tofali | Maurer |
| 26 | Duka | Laden | 81 | Kondakta | Dirigent | 136 | Mwandishi | Journalist | 191 | Tufaha | Apfel |
| 27 | Faharasa | Katalog | 82 | Koo | Kehle | 137 | Nafaka | Getreide | 192 | Tufani | Gewitter |
| 28 | Fataki | Rakete | 83 | Koti | Mantel | 138 | Ndege | Vogel | 193 | Tumbo | Magen |
| 29 | Fimbo | Hebel | 84 | Kucha | Nagel | 139 | Ndoo | Kübel | 194 | Ubao | Tafel |
| 30 | Flava | Musiker | 85 | Kulabu | Haken | 140 | Ngano | Weizen | 195 | Ubawa | Flügel |
| 31 | Forodhani | Flughafen | 86 | Kulisha | Futter | 141 | Ngazi | Treppe | 196 | Uga | Terrasse |
| 32 | Fulana | Weste | 87 | Kumbusho | Museum | 142 | Ngoma | Trommel | 197 | Ujumbe | Botschaft |
| 33 | Fundi | Handwerker | 88 | Kunya | Niederschlag | 143 | Ngozi | Leder | 198 | Ukanda | Korridor |
| 34 | Funguo | Schlüssel | 89 | Kupika | Köchin | 144 | Nguo | Uniform | 199 | Ukumbi | Halle |
| 35 | Gari | Motor | 90 | Kuruka | Fliege | 145 | Nguzo | Säule | 200 | Ukuta | Mauer |
| 36 | Gereji | Garage | 91 | Kuumia | Verletzung | 146 | Njia | Allee | 201 | Ulimi | Zunge |
| 37 | Gofu | Ruine | 92 | Lindi | Kanal | 147 | Njiwa | Taube | 202 | Unyasi | Rasen |
| 38 | Gumba | Daumen | 93 | Lori | Lastwagen | 148 | Nyota | Satellit | 203 | Uombi | Bewerbung |
| 39 | Habari | Radio | 94 | Mabao | Balkon | 149 | Nyundo | Hammer | 204 | Ushahidi | Zeugnis |
| 40 | Hati | Dokument | 95 | Madeski | Schreibtisch | 150 | Ofisa | Beamter | 205 | Ushairi | Dichter |
| 41 | Hatua | Stufe | 96 | Mafuta | Heizöl | 151 | Oga | Dusche | 206 | Uta | Bogen |
| 42 | Hekalu | Tempel | 97 | Mahewa | Klavier | 152 | Paka | Katze | 207 | Utenzi | Aktivist |
| 43 | Hospitali | Krankenhaus | 98 | Maji | Wasser | 153 | Pamba | Orden | 208 | Uwanja | Stadion |
| 44 | Irori | Benzin | 99 | Makaa | Kohle | 154 | Pazia | Vorhang | 209 | Vitunguu | Zwiebel |
| 45 | Jambazi | Räuber | 100 | Malkia | Königin | 155 | Picha | Fotograf | 210 | Wakodi | Mieter |
| 46 | Jangwa | Wüste | 101 | Mani | Wiese | 156 | Pikipiki | Motorrad | 211 | Walimu | Lehrerin |
| 47 | Jela | Gefängnis | 102 | Maua | Blume | 157 | Pombe | Alkohol | 212 | Waridi | Rose |
| 48 | Jengo | Gebäude | 103 | Mboga | Gemüse | 158 | Pua | Nase | 213 | Wasia | Testament |
| 49 | Jeraha | Wunde | 104 | Mbolea | Humus | 159 | Pumzikio | Villa | 214 | Wawindaji | Jäger |
| 50 | Jeti | Hubschrauber | 105 | Mbosho | Tasche | 160 | Pundamilia | Zebra | 215 | Wingu | Wolke |
| 51 | Jiko | Ofen | 106 | Mbunifu | Architekt | 161 | Punje | Hafer | 216 | Zahanati | Klinik |
| 52 | Joto | Heizung | 107 | Mchoraji | Maler | 162 | Rangi | Gemälde | 217 | Zana | Kamera |
| 53 | Jua | Sonne | 108 | Mchuma | Gewehr | 163 | Ridhe | Revolver | 218 | Zawadi | Geschenk |
| 54 | Jukwaa | Bühne | 109 | Mdomo | Lippe | 164 | Riwaya | Roman | 219 | Ziara | Gräber |
| 55 | Kadi | Einladung | 110 | Medali | Medaille | 165 | Rubani | Pilot | 220 | Ziwa | Schwimmbad |
